# Tetraspanin 8 (TSPAN 8) as a potential target for radio-immunotherapy of colorectal cancer

**DOI:** 10.18632/oncotarget.15787

**Published:** 2017-02-28

**Authors:** Aurelie Maisonial-Besset, Tiffany Witkowski, Isabelle Navarro-Teulon, Odile Berthier-Vergnes, Giovanna Fois, Yingying Zhu, Sophie Besse, Olivia Bawa, Arnaud Briat, Mercedes Quintana, Alexandre Pichard, Mathilde Bonnet, Eric Rubinstein, Jean-Pierre Pouget, Paule Opolon, Lydia Maigne, Elisabeth Miot-Noirault, Jean-Michel Chezal, Claude Boucheix, Françoise Degoul

**Affiliations:** ^1^ INSERM, U 1240, Clermont-Ferrand, France; ^2^ Université Clermont Auvergne, Imagerie Moléculaire et Thérapie Vectorisée, Clermont-Ferrand, France; ^3^ IRCM, Institut de Recherche en Cancérologie de Montpellier, Montpellier, France; ^4^ INSERM, U896, Montpellier, France; ^5^ Université Montpellier 1, Montpellier, France; ^6^ Université de Lyon 1, Lyon, France; ^7^ CNRS, UMR5534, Centre de Génétique et de Physiologie Moléculaires et Cellulaires, Villeurbanne, France; ^8^ CNRS/IN2P3, UMR6533, Laboratoire de Physique Corpusculaire (LPC), Clermont-Ferrand, France; ^9^ INSERM, UMR-S 935, Villejuif, France; ^10^ Université Paris-Sud 11, Orsay, France; ^11^ Université Paris Saclay, Saint-Aubin, France; ^12^ Gustave Roussy, Laboratoire de Pathologie Expérimentale, Villejuif, France; ^13^ INSERM U1071, Faculté de Médecine, Clermont Ferrand, France

**Keywords:** TSPAN8, radioimmunotherapy, colorectal cancer, dosimetry

## Abstract

Tetraspanin 8 (TSPAN8) overexpression is correlated with poor prognosis in human colorectal cancer (CRC). A murine mAb Ts29.2 specific for human TSPAN8 provided significant efficiency for immunotherapy in CRC pre-clinical models. We therefore evaluate the feasability of targeting TSPAN8 in CRC with radiolabeled Ts29.2. Staining of tissue micro-arrays with Ts29.2 revealed that TSPAN8 espression was restricted to a few human healthy tissues. DOTA-Ts29.2 was radiolabeled with ^111^In or ^177^Lu with radiochemical purities >95%, specific activity ranging from 300 to 600 MBq/mg, and radioimmunoreactive fractions >80%. The biodistribution of [^111^In]DOTA-Ts29.2 in nude mice bearing HT29 or SW480 CRC xenografts showed a high specificity of tumor localization with high tumor/blood ratios (HT29: 4.3; SW480-TSPAN8: 3.9 at 72h and 120h post injection respectively). Tumor-specific absorbed dose calculations for [^177^Lu]DOTA-Ts29.2 was 1.89 Gy/MBq, establishing the feasibility of using radioimmunotherapy of CRC with this radiolabeled antibody. A significant inhibition of tumor growth in HT29 tumor-bearing mice treated with [^177^Lu]DOTA-Ts29.2 was observed compared to control groups. *Ex vivo* experiments revealed specific DNA double strand breaks associated with cell apoptosis in [^177^Lu]DOTA-Ts29.2 treated tumors compared to controls. Overall, we provide a proof-of-concept for the use of [^111^In/^177^Lu]DOTA-Ts29.2 that specifically target *in vivo* aggressive TSPAN8-positive cells in CRC.

## INTRODUCTION

Communication between cells by direct or indirect contact is a key process regulating tissue development and homeostasis as well as recognition signals noticeably for cells involved in innate and adaptative immune responses. Tetraspanins, a family of membrane proteins with 4 transmembrane domains, are important actors in this process since these proteins can be found in both plasma membranes and exosomes [1]. Recent crystallographic structure of tetraspanin CD81 revealed a unique cone-like structure with an intramembrane pocket filled with a cholesterol molecule [2]. Remarkably, tetraspanins can control the function of some cell surface proteins including integrins and a variety of other proteins involved in cell-cell and cell-matrix interactions [3]. These intrinsic properties correlate with the involvement of Tetraspanins in several pathologies such as cancers and exogenous infections [4]. Among the 33 mammalian members of the Tetraspanins family, at least five of them, CD9, CD37, CD82, CD151 and TSPAN8, are reported to display altered expression in cancers when compared to normal tissues [1,5]. The pro- or anti- cancer properties of CD9 is still controversial, while CD151 and TSPAN8 are assumed to display oncogenic activities. On the contrary, CD82 acts as a unique metastasis suppressor [1,5]. Interestingly, CD37 protects against the development of B cell lymphoma by associating SOCS3 with IL-6 receptor and then blocking IL-6 pathway [6]. TSPAN8 also named Co-029 is expressed on epithelial cells of some healthy tissues (mainly stomach, ileum/jejunum, colon/rectum and liver) [7]. It is expressed at high level in tumor tissues like esophagus [8], stomach [9], colon [10], liver [11], pancreas [12], ovary [13], and neo-expressed in invasive cutaneous melanomas [14]. TSPAN8 is known to be positively correlated with the occurrence of metastases and bad prognosis [8,10,11]. Based on experimental data, several mechanisms were suggested to explain the protumoral properties of TSPAN8. Indeed, it has been shown to be involved in cell adhesion and motility due to its interaction with cell adhesion molecules [10,15–18], in invasiveness by increasing ADAM12m expression [8]. TSPAN8 also interferes with EGF signaling [9], induces diffuse coagulopathy [15] and promotes angiogenesis through exosomes secretion [12]. Furthermore,TSPAN8-specific antibodies reduce cell motility, angiogenesis, xenografted-tumor growth in nude mice and metastasis incidence [10,12,13,19]. TSPAN8 mRNA has been also recently identified as a highly sensitive and specific blood biomarker for colorectal cancer (CRC) detection [20]. Overall, these data argue that TSPAN8 could be viewed as a promising therapeutic target in human carcinomas.

One strategy to counteract the pro-metastatic role of Tetraspanins can be the targeting of signaling pathways downstream Tetraspanin partners like integrins or other receptors (*e.g*. small kinase inhibitors) [1]. Immunotherapy is an alternative approach relying on the use of monoclonal antibodies that can specifically recognize and target antigens expressed on tumor cells. This interesting strategy has been successfully developed to target mainly EGFR and VEGF in CRC with substantial responses [21,22]. Other antibodies were developed against IGFR (ganitumab) or acting as agonists of DR5 receptors (conatumumab) and tested in clinical trials without observed benefit [23]. Recently, a mouse monoclonal antibody Ts29.2 specific for human TSPAN8 was tested in preclinical models of CRC with cell lines expressing different levels of TSPAN8 [19]. A significant slowdown of the tumor growth was observed but the molecular mechanisms involved in this process are not yet fully elucidated.

The main drawback of immunotherapy is the poor penetration of antibodies into solid tumors including CRC. To circumvent this problem, conjugation of monoclonal antibodies to α or β^−^-emitting radionuclides can significantly improve their therapeutic effects. Indeed, local irradiation of tumor cells in the range of the emitted particles can also destroy neighboring cells exhibiting low antigen expression or non-accessible to antibodies. Typically, antibodies can be radiolabeled with iodine-131 (β^−^, E_max_=606 KeV, 8.03 days) on their tyrosine residues. Nevertheless, in the case of internalizing antibodies, this strategy can lead to a significant decreased dosimetry, due to metabolism generating free radioactive iodine or tyrosine radiometabolites that are easily excreted from cells [24]. Another interesting strategy to create more metabolically stable entities is the labeling of antibodies with radiometals such as yttrium-90 (β^−^, E_max_=2290 KeV, 2.67 days) or lutetium-177 (β^−^, E_max_=490 KeV, 6.73 days). In these cases, the radionuclide is introduced into the protein scaffold *via* conjugation of lysine or cysteine residues with chelating agents like DOTA. Such radioimmunotherapy (RIT) approaches have been largely developed with a radiolabeled anti-CD20 antibody (Zevalin®, [^90^Y]ibritumomab, tiuxetan) which is currently available for the treatment of lymphomas resistant to classical chemotherapy [25]. Despite [^90^Y]ibritumomab good efficiency, clinicians do not favor this approach due to practical issues such as nuclear department center availability, price, some bone marrow toxicity and also the development of other active treatments in lymphomas [26]. Interestingly, a CD37 antibody radiolabeled with lutetium-177 ([^177^Lu]betalutin) is currently under clinical evaluation (NCT01796171) for the management of patient with relapsed CD37^+^ non-Hodgkin lymphomas [27].

Although combination strategies recently developed in colon carcinomas allow substantial improvement in clinical outcome, there is an urgent need to discover new tumor-associated antigens as potential therapeutic targets. RIT approaches have also been tested in preclinical studies on CRC with significant effect alone or in association with either antibodies usually targeting transmembrane proteins (CEA, GPA33, EGFR) or chemotherapy [28–30]. Therefore, the main objective of this study was to assess the feasibility of targeting TSPAN8 in CRC with a radiolabeled antibody Ts29.2 for RIT purposes. To this end, we first examined the TSPAN8 tissue distribution pattern in a variety of normal human organs. Then, Ts29.2 was radiolabeled with indium-111 (γ, 171 and 245 KeV, 2.80 days) using DOTA as chelator to determine its biodistribution using planar scintigraphic gamma imaging in CRC preclinical mouse models (HT29 and SW480/SW480-TSPAN8 tumors engrafted on nude mice). Then, corresponding dosimetry parameters were calculated using the GATE Monte Carlo platform, in the HT29 model to predict the dose delivered to the tumor when using a β^−^-emitting radionuclide. Finally, the efficiency of the RIT using the antibody Ts29.2 radiolabeled with lutetium-177 was evaluated on HT29 CRC preclinical model by monitoring the tumor growth and investigating some molecular changes induced in tumors.

## RESULTS

### Ts29.2 detects TSPAN8 in a restricted number of human normal tissues

**Figure 1:**
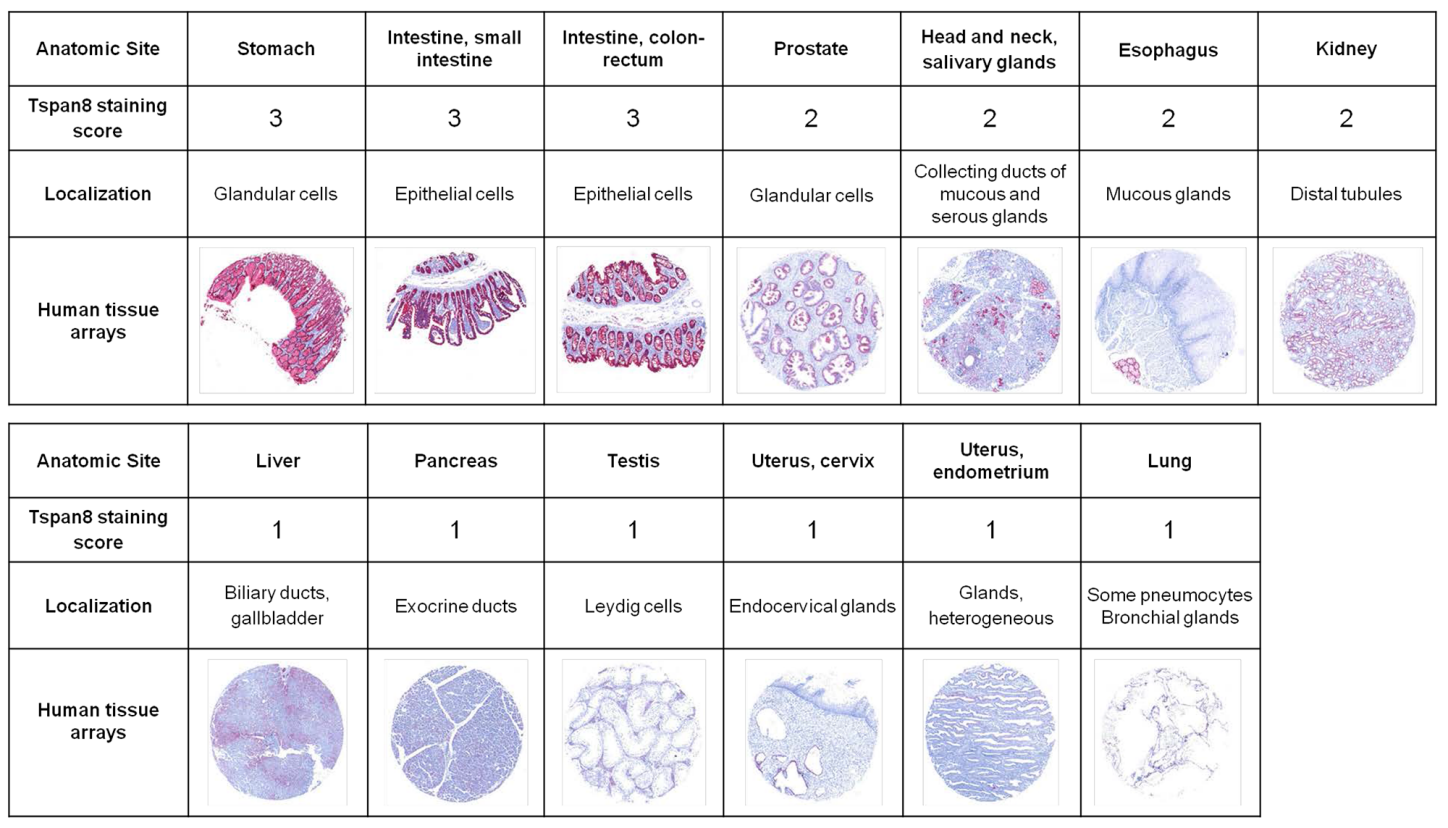
Immunoreactivity of Ts29.2 antibody in normal human organs Analysis of 34 different normal human tissue microarrays (Pantomics) using Ts29.2 antibody. All sections were stained with fast red and counterstained with hematoxylin. Selected images and representative scoring intensities were shown. Scoring 1, 2 and 3 corresponding to low, medium and high Ts29.2 staining.

We previously reported that Ts29.2 antibody targeting TSPAN8 slowed down the growth of human colon xenografts in nude mice [19], suggesting that TSPAN8 may represent a potentially attractive target for antibody-based therapy. Therefore, detailed specificity analysis of TSPAN8 expression in normal tissues is essential in the preclinical evaluation of Ts29.2. We thus performed an immunohistochemical study using a commercially available tissue microarrays (TMA) containing a broad panel of human normal tissues. Representative examples of TSPAN8 staining clearly showed that the most pronounced TSPAN8 expression (score 3) was found in only 3 of the 34 tissues examined: stomach, small intestine and colon (Figure 1). A moderate staining was observed in prostate, head and neck salivary glands, esophagus and kidney (score 2). A faint diffuse staining was seen in liver, pancreas, testis, uterus and lung (score 1). No other normal tissues had detectable TSPAN8 staining, including the adrenal gland, bladder, urine, bone, eye, breast, brain, fallopian tubes, heart, peripheral nerves, ureter, ovary, parathyroid, pituitary gland, placenta, skin, spinal cord, spleen, skeletal muscle, thymus, thyroid and tonsil (not shown). These data demonstrate that Ts29.2 antibody has a limited distribution in normal human tissues.

### Radiolabeling of DOTA-Ts29.2 with indium-111 and its biodistribution in preclinical models of colon carcinoma

**Figure 2:**
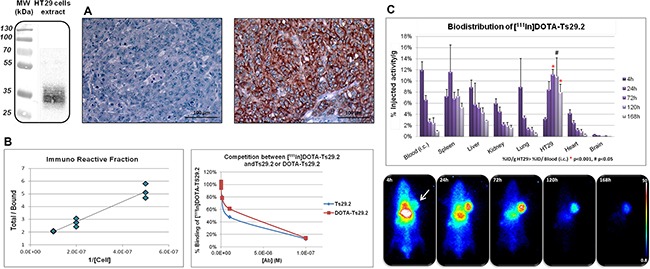
Specificity and biodistribution of [^111^In]DOTA-Ts29.2 in HT29 bearing mice **A.** Western blot (left part) and IHC analyses (right part) of TSPAN8 expression in HT29 cells and tumors respectively. Negative control on the left, labeling with Ts29.2 (TSPAN8) on the right **B.** Typical *in vitro* experiment of IRF determination with the linear regression analysis by plotting total/bound fraction vs. 1/[cell] (left part) and competition experiments of [^111^In]DOTA-Ts29.2 binding with increasing quantities of Ts29.2 or DOTA-Ts29.2 (right part). IRF was determined by linear regression (ax+b) and corresponded to 1/b value. **C.** Mice bearing HT29 tumors were injected (i.v.) with 3.7 MBq of [^111^In]DOTA-Ts29.2 and imaged with a γ-camera at 4 h, 24 h, 72 h, 120 h and 168 h post injection (lower part). Tumors, blood and organs were collected and weighted (3 mice/time point) and the radioactivity was measured by γ-counting of each sample. The graph represents the % of injected activity *per* gram of tissue (%IA/g) (upper part).

Ts29.2 was first conjugated with DOTA chelating moieties and purified *via* size exclusion semi-preparative HPLC. The number of DOTA *per* antibody ranged from 12 to 14 as determined by MALDI-TOF MS analyses. Further labeling of DOTA-Ts29.2 with indium-111 successfully provided [^111^In]DOTA-Ts29.2 with radiochemical yields >98%, specific activities (SA) of 544 MBq/mg or 304 MBq/mg and radiochemical purities of 99.8% and 95.0% for HT29 and SW480 studies respectively (See Supplementary Figure 1 left for Ts29.2). TSPAN8 expression in HT29 cells was then checked *in vitro* by western blot and *ex vivo* by IHC analyses (Figure 2A left and right, respectively). The immunoreactive fraction (IRF) tested on HT29 cells was 83 ± 8% (n = 4). A typical determination is shown in Figure 2B (left panel). The binding competition experiments between [^111^In]DOTA-Ts29.2 and Ts29.2 or DOTA-Ts29.2 gave similar results (Figure 2B, right panel). Biodistribution studies (Figure 2C) showed a specific accumulation of radioactivity in HT29 tumors, 72 hours after injection. This uptake was statistically different from the blood content from day 3 to day 5. In all other organs except spleen the average percentage of injected radioactivity (%IA) of [^111^In]DOTA-Ts29.2 decreased in parallel to the %IA in the blood. The spleen can fix the Fc region of the antibody leading to nonspecific binding [32].

**Figure 3:**
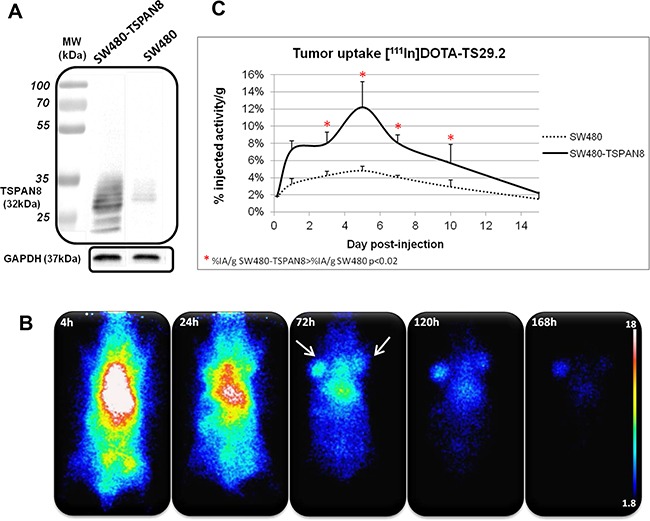
Specific biodistribution of [^111^In]DOTA-Ts29.2 in SW480 and SW480-TSPAN8 bearing mice Difference in TSPAN8 expression was assessed by western blot analysis in the two cell lines, SW480 expressing faint basal level of TSPAN8 and transduced SW480-TSPAN8 expressing high levels of TSPAN8 **A.** Mice bearing both tumors (SW480 on the right side and SW480-TSPAN8 on the left side) were injected (i.v.) with 3.7 MBq of [^111^In]DOTA-Ts29.2 and imaged with a γ-camera at 4 h, 24 h, 72 h, 120 h and 168 h post injection **B.** Tumors, blood and organs were collected and weighted (3 mice/time point) and the radioactivity was measured by γ-counting of each sample. The graph represents the % of injected activity per gram of tissue (%IA/g) **C**.

Similar experiments were performed with SW480 and SW480-TSPAN8 cell lines (Figure 3). This model allowed us to confirm the specificity of [^111^In]DOTA-Ts29.2 toward TSPAN8. A clear correlation between the content of TSPAN8 in the cells and the %IA in tumors was found. The binding specificity was then confirmed by the statistically significant difference in uptake between the two types of cell lines.

### Dosimetry calculations

The estimated doses of [^177^Lu]DOTA-Ts29.2 or [^90^Y]DOTA-Ts29.2 delivered to tumor, kidney, liver, lungs and brain were summarized in Table 1. The computed S-factors were listed in Supplementary Tables 1 and 2. S-factor values for [^90^Y]DOTA-Ts29.2 were higher than for [^177^Lu]DOTA-Ts29.2 due to the higher average energy of β^−^ particles emitted by yttrium-90 with respect to the ones emitted by lutetium-177 (930 keV compared to 149 keV respectively). As the DOTA chelating moiety can be used for both radiolabeling with indium-111, lutetium-177 or yttrium-90, the cumulated activities for [^177^Lu]DOTA-Ts29.2 and [^90^Y]DOTA-Ts29.2 were extrapolated from the [^111^In]DOTA-Ts29.2 biodistribution results. The calculated dose delivered to the tumor was 1.5 higher with [^90^Y]DOTA-Ts29.2 compared to [^177^Lu]DOTA-Ts29.2, in accordance with the physical properties of yttrium-90. However, the ratio of absorbed doses in the tumor to non-targeted organs (Table 1) was higher with [^90^Y]DOTA-Ts29.2 compared to [^177^Lu]DOTA-Ts29.2. Consequently, the RIT protocol was performed using the radiolabeled conjugated [^177^Lu]DOTA-Ts29.2.

**Table 1:** Dosimetry calculations performed by computing S factors with the GATE Monte Carlo platform and biodistribution data. Dose values (Gy) were calculated for a 3.7 MBq injection of the radiolabeled antibody Ts29.2

	Dose (Gy)		Dose_Tumor_/Dose_Organ_	
	^177^Lu	^90^Y	^177^Lu	^90^Y
**Tumor**	7.00 ± 2.00	11.0 ± 3.0	_	_
**Spleen**	3.70 ± 0.30	8.9 ± 0.7	1.89	1.24
**Kidneys**	0.96 ± 0.09	11.0 ± 1.0	7.29	1.00
**Liver**	2.70 ± 0.20	9.6 ± 0.6	2.59	1.15
**Heart**	0.34 ± 0.05	5.4 ± 0.7	20.59	2.04
**Lungs**	0.45 ± 0.09	7.0 ± 1.0	15.56	1.57
**Brain**	0.16 ± 0.01	4.9 ± 0.2	43.75	2.24
**Rest of body**	1.70 ± 0.10	9.5 ± 0.8	4.12	1.16

Ratios of doses between tumor and other organs were also evaluated

### Radioimmunotherapy experiments

The radiolabelings of the DOTA-Ts29.2 or DOTA-16F12 (irrelevant antibody) conjugates with lutetium-177 were performed as previously described for indium-111. [^177^Lu]DOTA-Ts29.2 and [^177^Lu]DOTA-16F12 were obtained with radiochemical yields >97%, specific activities of 346 MBq/mg and 349 MBq/mg and radiochemical purities of 99.4% and 95.4%, respectively (for Ts29.2 Supplementary Figure 1 right).

**Figure 4:**
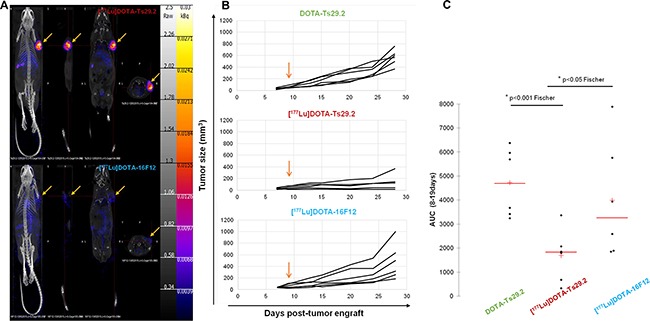
Radioimmunotherapy using [^177^Lu]DOTA-Ts29.2 in HT29 bearing mice **A.** SPECT-CT imaging of mice bearing HT29 tumors 5 days after receiving i.v. injections of [^177^Lu]DOTA-Ts29.2 (upper part) or [^177^Lu]DOTA-16F12 (lower part) treatments. Arrows indicate the HT29 tumors. **B.** Mice received different i.v. injections 9 days post HT29 graft (arrow), the tumor volume was reported for each mice for 28 days after engrafts (n = 6 per group). **C.** Evaluation of tumor volume using the areas under the curve from data obtained in B.

The mean of tumor volume the day before treatment start was similar between the three groups. The tumor growth of DOTA-Ts29.2 treated control mice was not different from those of mice receiving only saline solution (data not shown). In mice receiving [^177^Lu]DOTA-Ts29.2, the tumor volumes were significantly lower than those of animals receiving DOTA-Ts29.2 or those treated with a non-specific RIT ([^177^Lu]DOTA-16F12, Figure 4B). The areas under the curve determined for each mice, were significantly lower in [^177^Lu]DOTA-Ts29.2 group than in non-radiolabeled DOTA-Ts29.2 or [^177^Lu]DOTA-16F12 groups (Figure 4C). Furthermore, five days post-treatment, SPECT/CT quantification revealed a tumor/muscle (T/M) ratio of 18.40 and 2.20 for specific and non-specific RIT respectively (Figure 4A). There was no variation between the body weights of the different groups (data not shown).

**Figure 5:**
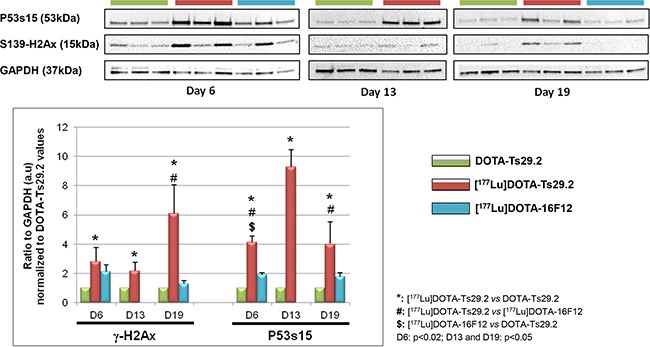
Molecular studies on HT29 tumors The phosphorylation of P53 on serine 15 and of H2AX histone on serine 139 was monitored by western blot analyses in three mice at days 6 and 19 for each group and at day 13 for DOTA-Ts29.2 and [^177^Lu]DOTA-Ts29.2 groups (upper part). The level of phosphorylated proteins was normalized by GAPDH as a loading control (lower part).

**Figure 6:**
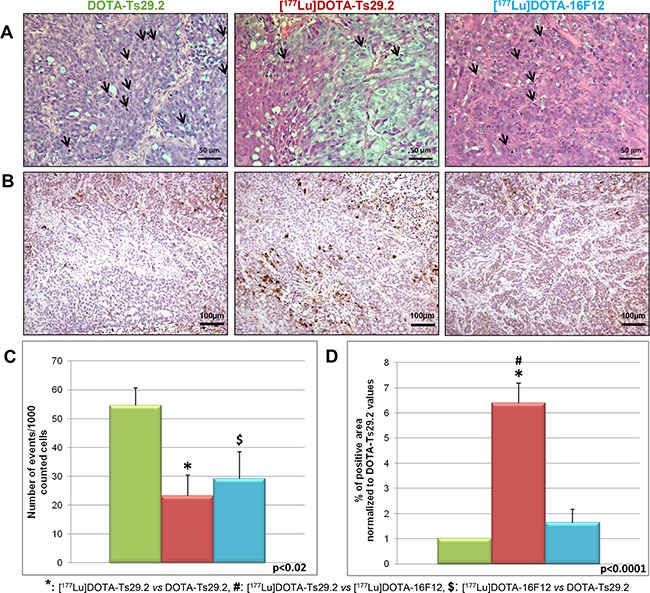
Immunohistochemical analyses of HT29 tumors performed 6 days following treatments HES allowed to observe **A.** and quantify cells undergoing mitosis **C.** The mitotic cells are marked by arrows (A). Three tumors were analyzed for each group and two fields were counted corresponding to at least 300 analyzed tumor cells per field. The Activity of caspase was assessed by quantification of cleaved caspase 3 revealed by IHC **B.** The surface of labeled cells was calculated for three tumors in each group and reported to the total surface to give the % of apoptotic areas **D.** Scale bars represent 50 μm (HES) and 100 μm (IHC).

Examination of the phosphorylation status of p53(S15) and H2AX(S139) in tumors revealed that [^177^Lu]DOTA-Ts29.2 treatment induced specific and long-term effects on DNA double-strand breaks (DSB) concomitantly with signs of death (Figure 5). The presence of DSB (γ-H2AX) was transitory when using the non-specific RIT (day 6) and remained detectable until day 19 only for the group which received a dose of [^177^Lu]DOTA-Ts29.2. p53 phosphorylation on serine 15 followed the same pattern. In addition 6 days post-treatment, the percentage of mitotic cells was significantly lower in [^177^Lu]DOTA-Ts29.2 treated tumors compared to non-radiolabeled DOTA-Ts29.2 (Figure 6A and 6C). A significant increase in the number of apoptotic cells was also shown by immunohistochemistry for cleaved caspase 3 in tumors which received the specific RIT compared to the other groups (Figure 6B and 6D).

## DISCUSSION

Various monoclonal antibodies targeting a broad array of cell-surface antigens that are overexpressed, mutated or selectively expressed compared with normal tissues have been explored in CRC. Here, we address the feasibility of RIT approach by targeting a novel tumor specific antigen: TSPAN8, using the monoclonal antibody Ts29.2 [19]. Indeed, it has been previously shown to exhibit anti-tumor effects on xenograft models. A prerequisite for successful RIT is that TSPAN8 has a limited expression in most vital organs. We found a strongest Ts29.2 reactivity exclusively in the digestive epithelial cells of stomach, small intestine and colon, which was consistent with immunohistochemistry-based protein profiling data available from the Human Protein Atlas (http://www.proteinatlas.org/ENSG00000127324-TSPAN8/tissue). Even though a moderate staining was found in 4 of 35 healthy organs (salivary gland, esophagus, kidney, prostate), we can conclude that 28 of *35* (80%) organs are negative. In human colon carcinomas, Greco *et al*. (2010) have found a strong Ts29.2 labeling in tumor areas compared to normal neighboring epithelium. This corroborates with an extensive RNA-seq study where TSPAN8 mRNA was found highly expressed in cell lines from colon and stomach carcinomas (Supplementary Figure 2) [33]. The validation of this antibody was then effective as a specific radiotracer for both imaging and therapy of CRC tumors.

Indium-111 was chosen for its suitable half-life (2.80 days) and physical properties (γ, 171 and 245 KeV) allowing *in vivo* biodistribution studies *via* imaging with radiolabeled antibodies which usually have a long biological half-life. In addition, the use of such radiometal allows convenient radiolabeling protocols *via* complexation with macrocyclic DOTA chelator. These methods can be easily adapted for the radiolabeling with therapeutic radioisotopes such as yttrium-90 or lutetium-177. Accordingly, chelating moieties were firstly introduced on the Ts29.2 antibody using conjugation with activated DOTA-NHS in a ratio 200/1, antibody/chelate. A large excess of DOTA-NHS was used to offset the fast hydrolysis of the activated ester function in aqueous media (pH>7). Following such protocol, an average number of 12-14 DOTA were grafted on the protein scaffold, as determined by MALDI-TOF mass spectroscopy analyses. Then, classical radiolabeling with indium-111 was realized by incubation of the DOTA-Ts29.2 conjugate with [^111^In]InCl_3_. In these conditions, high radiochemical yields (RCY >98%), radiochemical purities (RCP >95%) and specific activities (SA>300 MBq/mg) were achieved. The immunoreactive fraction of 83 ± 8% as the competition experiments (Figure 2B) highlighted that the addition of 12-14 chelating moieties on the Ts29.2 scaffold did not induce major modifications in the antibody binding capacity to TSPAN8 *in vitro* and proved the suitability of [^111^In]DOTA-Ts29.2 for further *in vivo* biological evaluations.

[^111^In]DOTA-Ts29.2 biodistribution in nude mice clearly supported its specificity for human CRC tumors expressing TSPAN8 with a significant differential uptake of radioactivity between basal SW480 and SW480-TSPAN8 in agreement with the difference of TSPAN8 protein levels (Figure 3). Nonspecific binding was mainly observed in the spleen, due to interaction of the Fc region of the antibody with receptors of macrophages and lymphoid cells subsets [32]. In other organs, the radioactivity content decreased for nonspecific binding, as expected (Figure 2). [^111^In]DOTA-Ts29.2 biodistribution in HT29 model was used to perform dosimetry of the corresponding lutetium-177 or yttrium-90 conjugate. The calculated doses (Table 1) confirmed the feasibility of a RIT approach using [^177^Lu]DOTA-Ts29.2 with values of delivered dose to tumors comparable to those obtained for [^177^Lu]HH1 and [^177^Lu]rituximab [27]. In addition, the delivered dose to non-target organs was significantly lower when using [^177^Lu]DOTA-Ts29.2 compared to [^90^Y]DOTA-Ts29.2. Taken together, these results led to the selection of lutetium-177 as radionuclide to perform our first RIT experiment with Ts29.2.

After successful radiolabeling with lutetium-177 (RCY >97%; RCP >99%; SA>346 MBq/mg), the injection of [^177^Lu]DOTA-Ts29.2 induced a significant slowdown of HT29 tumor growth compared to the use of the non-specific antibody [^177^Lu]DOTA-16F12 with 2 out of 5 mice receiving [^177^Lu]DOTA-Ts29.2 having a tumor which did not grow at all. However, a slight decrease of tumor growth was also observed using [^177^Lu]DOTA-16F12. Although we ruled out the presence of the anti-Mullerian hormone receptor in HT29 models by CMF (data not shown), we cannot exclude that some binding to unmasked antigen occurred *in vivo*. Indeed, the SPECT-CT images revealed that some [^177^Lu]DOTA-16F12 fixation occurred in HT29 xenografts (specific *vs*. non specific T/M ratio of 8.45). Another possibility to explain the [^177^Lu]DOTA-16F12 fixation relies on the enhanced permeability and retention effect (EPR effect) due to altered tumor vessels properties [34].

Molecular studies on HT29 tumors demonstrated that [^177^Lu]DOTA-Ts29.2 induced a classical response to radiotherapy with phosphorylation of p53 on serine 15 and of histone H2AX on serine 391, leading to apoptotic cell death, highlighted by increased caspase 3 activity (Figures 5 and 6). An associated decrease of the number of cells undergoing mitosis was also demonstrated. In the case of the [^177^Lu]DOTA-16F12 treatment, phosphorylation of p53 and H2AX can also be observed but only in the first days following RIT. No activation of caspase 3 can be retrieved despite a significant decrease of the number of mitoses in the [^177^Lu]DOTA-16F12 treated tumors. Similar molecular analyses with high quantity of non-radioactive Ts29.2 failed to detect cell death in treated tumors [19] showing the role of radiations in DSB occurrence and cell death.

Considering secondary effects of the RIT protocol in this model, the pattern of distribution of [^111^In]DOTA-Ts29.2/[^177^Lu]DOTA-Ts29.2 showed a classical long half-life in blood, and no specific retention in normal tissues, except in spleen where Fc regions of the IgG can bind Fc receptors on splenic cells. Should TSPAN8 be selected for RIT in humans, modified antibodies would be designed to avoid or reduce non-specific binding. Several studies reported the presence of TSPAN8 in the blood as a component of tumor-derived exosomes [35] or as candidate mRNAs for detection of CRC [20]. A solution to limit blood toxicity due to specific binding and to long half-life of antibodies can be the use of pretargeting strategies [36]. This approach, currently under investigation in our laboratory, reduces blood toxicity while allowing injecting higher quantity of radioactivity to increase the dose delivered to the tumors. The human tumor xenograft model using immunocompromised mice without metastasis occurrence can be considered unsatisfactory. Therefore, we are currently evaluating an antibody designed for the recognition of murine TSPAN8 in *Apc^Min/+^* mice that develop multiple adenomas in their small intestine and colon that advance to more tumors in old animals [37]. We hypothesize that targeting TSPAN8 by RIT will reduce the ability of aggressive TSPAN8^+^ cells to invade adjacent tissues or colonize distant organs.

In conclusion, Ts29.2 has been successfully radiolabeled for imaging and RIT in mice bearing CRC xenografts. The data collected can support the development of an humanized Ts29.2 IgG_2_ antibody to further evaluate its efficacy in a clinical trial. TSPAN8 should be considered as a promising target to fight not only aggressive CRC tumors, but also a broad range of other cancer types, such as melanoma [14], glioma [38], hepatocellular carcinoma [39], ovarian [13] and gastric cancers [40].

## MATERIALS AND METHODS

### Antibodies and reagents

The mouse IgG2b antibody specific for human TSPAN8 (TS29.2) was obtained from a BALB/c mouse hybridoma supernatant culture, produced in the laboratory of Dr Claude Boucheix (Paris, France) and purified as previously described [19]. This antibody has been previously shown to slow down growth of colon xenograft tumors in nude mice [19]. A non-target, irrelevant IgG2b antibody (16F12) directed against the human Mullerian inhibiting substance type II receptor [41] was provided by Dr Isabelle Navarro-Teulon (Montpellier, France). 2,2′,2′’-(10-(2-((2,5-dioxopyrrolidin-1-yl)oxy) -2-oxoethyl)-1,4,7,10-tetraazacyclododecane-1,4,7-triyl)triacetic acid (DOTA-NHS) was purchased from CheMatech (Dijon, France). [^111^In]InCl_3_ in 0.05 M HCl solution and [^177^Lu]LuCl_3_ solutions were purchased from Mallinckrodt Pharmaceuticals (Chesterfield, Derbyshire, United Kingdom) and PerkinElmer Life and Analytical Sciences (Waltham, MA 02451, USA) respectively. Size exclusion HPLC analyses were performed on a Superose 12 column, 10/300 GL, 11 μm (GE Healthcare), isocratic elution (20 mM 4-(2-hydroxyethyl)-1-piperazineethanesulfonic acid HEPES, 150 mM NaCl, pH = 7.3, flow rate: 0.5 mL.min^−1^) using a Perkin Elmer system equipped with a Flexar LC autosampler, a Series 200 pump, a Peltier column oven, a vacuum degasser, a Photodiode Array Detector (PDA) and a GabiStar detector (Raytest). Determination of the antibody concentrations were performed on a NanoDrop spectrophotometer (Thermoscientific, MultiskanGo). Matrix-assisted laser desorption/ionisation–time of flight–mass spectrometry (MALDI-TOF-MS) analysis was performed on a Voyager DE-PRO mass spectrometer (Applied Biosystems, Framingham, MA, USA).

### Tissue microarray

Commercially available adult human normal tissue microarrays (TMAs) were purchased from Pantomics (Pantomics, Inc., San Francisco, CA, USA; catalog number MNO1021). They contain 102 spots (1.5 mm diameter) of the following 34 tissue types (3 spots for each): adrenal gland, bladder, brain, breast, bronchus, bone, cerebellum, cerebrum, colon, diaphragm, fallopian tube, esophagus, heart, kidney, liver, lungs, lymph node, ureter, ovary, pancreas, prostate, skeletal muscle, skin, small intestine, spleen, stomach, testis, thymus, uterus, thyroid, pituitary gland, spinal cord, tonsil. TSPAN8 staining was carried out with mouse monoclonal antibody (Ts29.2; 1/2000) as previously described [14]. Negative controls were conducted by replacing the primary antibody with preimmune mouse serum. Staining intensity was quantified as follows: 0 as negative, 1 as weak, 2 as moderate, and 3 as strong. All images were captured using the VENTANA iScan HT slide scanner (Roche Diagnostics).

### Cell lines and culture

HT29 and SW480 colorectal carcinoma cell lines were obtained from ATCC. SW480-TSPAN8 was previously obtained by transduction with a lentiviral vector containing the coding region of the tetraspanin 8 [19]. Cell lines were cultured in Dulbecco’s modified Eagle’s medium (DMEM) supplemented with 10% FCS, glutamax and antibiotics (all purchased from Invitrogen). The cells were maintained at 37 °C in an atmosphere of 5% CO_2_ in air and all the incubations with radiolabeled antibodies were performed under the same conditions.

### Conjugation of antibodies with DOTA-NHS and radiolabeling with indium-111 or lutetium-177

Typically, 2 mg of Ts29.2 or 16F12 antibodies (29 μL, 42 mg/mL in PBS) were added to 2 mg of DOTA-NHS ester freshly introduced in a low retention tube (ratio DOTA-NHS/antibody: 200/1). The resulting solution was diluted with DPBS buffer (600 μL) and the pH of the reaction mixture was adjusted to 7.2-7.4 by portionwise addition of a 0.1 M aqueous sodium hydroxide solution (100-130 μL). The resulting mixture was stirred (end-over-end rotation) overnight at 4 °C. After return back to room temperature, the reaction mixture was transferred on an Amicon® Ultra centrifugal filter (50K, Millipore) and the tube was rinsed twice with 1 mL of Milli Q water. The resulting diluted reaction mixture was concentrated by centrifugation to less than 100 μL and purified by semi-preparative size exclusion HPLC. The fractions containing the DOTA-Ts29.2 or DOTA-16F12 conjugate were collected (3-4 mL; R_t_: 20.1 min for DOTA-Ts29.2; R_t_: 19.8 min for DOTA-16F12) and concentrated to less than 100 μL by centrifugation using an Amicon® Ultra centrifugal filter (50K, Millipore). The final concentration was estimated using Nanodrop and the number of grafted DOTA *per* antibody was determined using MALDI-TOF mass spectrometry.

For radiolabeling, 2 μL of the previously concentrated solution of DOTA-Ts29.2 or DOTA-16F12 conjugates (22.7 μg/μL) were diluted with 0.1 M HEPES buffer (pH 5.5, 248 μL). Then, a solution of [^111^In]InCl_3_ or [^177^Lu]LuCl_3_ in 0.05 M HCl (60 μL, 17 MBq) was added and the reaction mixture was vortexed before incubation at 45 °C for 15-20 min. The radiochemical purities of resulting [^111^In]DOTA-Ts29.2, [^177^Lu]DOTA-Ts29.2 or [^177^Lu]DOTA-16F12 were determined using size exclusion analytical radio-HPLC analyses.

### [^111^In]DOTA-Ts29.2 immunoreactivity to HT29 cells

Different quantities of HT29 cells (1.10^6^, 2.10^6^, 5.10^6^ and 10.10^6^) were incubated in a binding media (25 mM HEPES pH = 7, containing 0.2% of bovine serum albumin (BSA), completed with modified Eagle’s medium (MEM) with 0.6 pmol/L of [^111^In]DOTA-Ts29.2 (37 kBq/L) for 30 min under gentle shaking. Then, samples were centrifuged at 1000 g for 8 min at room temperature. The supernatant was removed, the cell pellets were washed with PBS containing 0.2% of BSA and centrifuged again at 1000 g for 8 min. The supernatant was again removed. The radioactivity in both supernatants (S) and pellets (B) from each sample was measured using a γ-counter (Wallac 1480 Wizard® 3‘‘, Perkin Elmer) to determine the percentage of [^111^In]DOTA-Ts29.2 bound to HT29 cells. The total radioactivity (T) was calculated by adding B and S fractions. The immunoreactive fraction (IRF) was calculated by plotting T/B *vs*. 1/[cell concentration] and with a linear regression analysis [31] and corresponds to the inverse value of the origin ordinate. This parameter is based on the calculation of the saturating antigen concentration required for the binding of a given antibody and extrapolation to conditions representing an infinite antigen excess (as described by Lindmo *et al*, [31]). The “Lindmo” assay is based on a double-inverse plot of the binding data which may be considered a modification of the Lineweaver-Burk plot.

### Binding competition experiments

HT29 cells (25000 cells *per* well) were seeded in a 24 multi-well dish in complete medium. After 24 hours, the supernatant was removed and the cells were washed gently with PBS. In each well, 300 μL of the competition media (25 mM HEPES pH = 7, containing 0.2% BSA, completed with MEM) containing 1.85.10^−3^ MBq of [^111^In]DOTA-Ts29.2 were added. For competition, Ts29.2 or DOTA-Ts29.2 from 0 to 1.10^−7^ M was included in the reaction media. After 1 hour of incubation under gentle shaking, the supernatant was removed and each well was washed gently twice with PBS containing 2% of BSA. The radioactivity was recovered using 400 μL of an aqueous 1 N NaOH solution. The radioactivity in pooled supernatants and washings as well as in cell fractions was measured using a γ-counter (Wallac 1480 Wizard® 3‘‘, Perkin Elmer). The percentage of binding was calculated taking into account the value obtained in the absence of non-radioactive antibody.

### *In vivo* experiments: xenografts and tumor growth

#### Animal models

Animal studies were performed according to protocols approved by local ethical committee. NMR1 Foxn1nu/Foxn1nu female mice were obtained from Janvier Labs (Le Genest-Saint-Isle, France) and used for biodistribution and RIT studies (animal’s weight: 24.5 ± 1.3 g and 27.6 g ± 1.4 g respectively). For these xenografts, cell suspensions were rinsed and diluted in 100 μL of PBS before injection. Mice were injected subcutaneously with 5.10^6^ or 3.10^6^ HT29 colon carcinoma cells in the shoulder on day 0 (respectively for biodistribution and RIT studies). In the same way, mice were injected with 5.10^6^ of SW480 and SW480-TSPAN8 colon carcinoma cells in right or in left shoulder respectively. Tumor volume was measured twice a week with a caliper by the same operator. Appearance and body weight of animals were also monitored with respect to ethical rules.

#### Biodistribution studies

22 and 20 days after the graft, for HT29 and SW480 models respectively, mice were injected intravenously with 3.5 ± 0.4 MBq of [^111^In]DOTA-Ts29.2 *per* mouse and randomly divided in 5 groups corresponding to the time points of the study (4 h, 24 h, 72 h, 120 h and 168 h post injection). Three mice *per* time point were sacrificed after chemical anesthesia (ketamin 100 mg/kg – Imalgen 500®, Merial and xylazine 10 mg/kg – Rompun® 2%, Bayer) followed by cervical dislocation. The mice of the last sacrificed group (i.e. 168 h p.i.) were imaged at each time point using a γ-camera (γ IMAGER, BIOSPACE Inc.) under gaseous anesthesia (Isoflurane, Iso-Vet® 1000 mg/g). For all groups, samples of blood (*via* cardiac puncture), tumor and several organs were collected, weighted and counted with a γ-counter (Wallac 1480 Wizard® 3‘‘, Perkin Elmer). Results are represented as a percentage of injected activity *per* gram of tissue (%IA/g).

#### Scintigraphic imaging

Planar scintigraphic imaging (10 minutes of acquisition) was performed on γ-camera (γ-acquisition BIOSPACE, France) using parallel collimator (20 mm/1.54/0.6) and signals were detected for indium-111 in a range of 149-190 keV and 210-259 keV. Image treatments were performed with γ-camera software (γ-vision+, BIOSPACE, France).

#### Dosimetry calculations

Dosimetry was established using the MIRD methodology [42]. The absorbed dose is calculated by multiplying the cumulative activity (Bq.s/kg) in the organ with the so-called “S-value”, representing the energy deposited in the organ *per* decay (J/Bq/s). First, activity over time from 4 h to 168 h post injection for each organ was fitted by a mono-exponential function except for tumor and spleen for which polynomial and mono-exponential were both used. Each cumulated activity to organs was then calculated with the Octave 4.0.2 software using “quad” function that performs numerical integration based on Gaussian quadrature. S-factors were calculated using the GATE Monte Carlo platform version 7.1 [43]. Three materials were defined according to the ICRP Publication 89 [43]: soft tissue (1.04 g·cm^−3^), lungs (0.296 g·cm^−3^), and bone (1.4 g·cm^−3^). Geometry was built from a mouse CT-scan and segmentation was performed with ISOgray Software by DOSIsoft [44]. The mice bearing HT29 tumors were injected with ExiTron (Myltenyi Biotec) and CT scans were performed using an Explore CT 120® (GE Healthcare) (Supplementary Figure 1). For each organ, a simulation was performed to compute S-value using uniform distribution of radioisotopes as sources (Supplementary Tables 1 & 2). Beta spectra were used to define source energy. Each simulation was performed with 10^9^ source particle to ensure a relative statistical uncertainty below 1%. Physical processes were simulated according to the electromagnetic physics-list emstandard_opt3 designed for application requiring higher accuracy for electrons. Production threshold value was set to 20 μm for electrons. Absorbed dose to the target was then calculated as the product of cumulated activity with corresponding S-factor.

#### Radioimmunotherapy experiments

9 days after the tumor grafts, mice were randomly divided in 3 treatment groups (15 animals *per* groups) and injected intravenously with: DOTA-Ts29.2 (15 μg/mouse) as non-radioactive control, [^177^Lu]DOTA-16F12 (7.0 ± 0.5 MBq/mouse) as non-specific RIT or [^177^Lu]DOTA-Ts29.2 (6.5 ± 0.6 MBq/mouse) for specific RIT. Animals were sacrificed after gaseous anesthesia overdose (Isoflurane, Iso-Vet® 1000 mg/g) followed by cervical dislocation 6, 13 and 19 days post treatment. The tumor volume was measured with a caliper by the same operator twice a week during all the experiment on the group of mice sacrificed 19 days post treatment. Then, tumors were collected and divided in two parts: one was fixed in A.F.A. (Diapath, MicromMicrotech, France) and the other one was frozen at -80 °C for further analyses.

#### SPECT/CT imaging

5 days following treatment injection, 3 animals (2 with specific RIT and one with non specific RIT) were imaged using a small animal SPECT Imaging camera (NanoSPECT-CT Plus; BIOSCAN, Washington, DC, USA) and whole body SPECT/CT tomographic images were acquired with multi pinhole collimators. Briefly, mice were place under gaseous anesthesia and SPECT/CT images were acquired sequentially using Nucline software (Mediso, Hungary). Co-registration of images was carried out using InVivoScope 2.0 software (inviCRO, USA). Quantification analyses were performed with VivoQuant 2.5 software (inviCRO, USA).

#### Immunohistochemistry analyses

Tissues removed from nude mice, were fixed in A.F.A. (Diapath, MicromMicrotech, France) and embedded in paraffin using the Thermo Scientific™ STP 120 Spin Tissue Processor. Paraffin sections (4 μm thick) were prepared on a microtome (Microm HM 340E Rotary Microtome, Thermo Scientific). After treatment with xylene solution (SafeSolv Q Path®, VWR International, USA) and rehydratation using standard protocol, routine Hematoxylin Eosin Saffranin (HES) staining was performed, allowing mitosis counting. For TSPAN8 detection, enzymatic induced antigen retrieval was achieved using the Protease I® solution (Ventana Medical System, Inc., Roche) 10 min at 37 °C. Sections were then incubated with blocking buffer (PBS-10% SVF-2% BSA) for 10 min at room temperature. Indirect staining was carried out by incubating the sections overnight at 4 °C with Ts29.2 at 10 μg.mL diluted in PBS-1% BSA. Then, sections were incubated 1 hour with the HRP Goat anti-mouse IgG (1:1000, Southern Biotech). The complex was revealed with the DAB substrate (Thermo Scientific), counter stained with Harris’s Hematoxylin and mounted (Eukitt®, Chem-Lab, Belgium). The level of cleaved caspase 3 was assessed by immunohistochemistry using antibody (1:1000, Cell Signaling) and revealed by peroxidase/diaminobenzidine Rabbit PowerVision kit (ImmunoVision Technologies).

### Protein extraction and western blotting

#### *In vitro* analyses

Subconfluent HT29 cell monolayers were washed with PBS and detached from the flasks by Trypsine-EDTA solution (Gibco®). Cells were lysed for 10 min at 4 °C with RIPA buffer (25 mM HEPES pH = 7, 15 mM NaCl, 1 mM EDTA, 1% Triton, 0.2% SDS, 0.5% sodium desoxycholate), and supplemented with protease inhibitors (Protease inhibitor cocktail tablets, Roche®).

Protein concentration was determined in supernatants after centrifugation (10 min, 1800 g, 4 °C) using Coomassie reaction at 595 nm. Proteins (50 μg) were separated on 4-15% SDS PAGE (Mini Protean® TGX gels, BIO-RAD) under non-reducing conditions and then transferred to nitrocellulose membranes (45 μm, Biorad). After blocking (5% non-fat milk), immunoreaction was performed with the Ts29.2 antibody (10 μg.mL) followed by horseradish peroxidase-labeled anti-mouse secondary antibodies (1:5000, Southern Biotech). Revelation was performed using the enhanced chemiluminescence detection system (Amersham™, ECL ™ Western Blotting detection Reagent, GE Healthcare), analyzed with an imaging system (ChemiDoc™ XRS+ System, Biorad), and quantified using the Image Lab software (Biorad).

#### *Ex-vivo* analyses

Tissues removed from nude mice at different post-treatment time points were hard frozen in the liquid nitrogen and stored at −80 °C. Total proteins were extracted from tissues by GentleMax tissue disruption in urea buffer (6 M Urea, 5 mM sodium fluoride, 2.5 mM sodium pyruvate, 1 mM EDTA, 0.5% Triton, 1 mM sodium orthovanadate) supplemented with protease inhibitors (Protease inhibitor cocktail tablets, Roche®).

Protein concentration determination, separation under reducing conditions and revelation were realized as described above. Immunoblotting was done with the indicated antibodies; Anti-Phospho-p53 (Ser15) Cell signaling (#9284), Anti-Phospho-Histone H2AX (Ser139) Cell Signaling (#2577), Anti-GAPDH Santa Cruz (FL-335: sc-25778) followed by corresponding horseradish peroxidase-labeled secondary antibodies (Southern Biotech). Development, imaging and relative quantification were performed as described above.

### Statistical analyses

Results were analyzed for statistical significance using the ANOVA parametric test for variance comparison, with p<0.05 as the statistically significant value (Excel Stat software). Data are presented as mean + SEM.

## SUPPLEMENTARY MATERIALS FIGURES AND TABLES



## Abbreviations

DOTA: 2,2′,2′’-(10-(2-((2,5-dioxopyrrolidin-1-yl)oxy)-2-oxoethyl)-1,4,7,10-tetraazacyclododecane-1,4,7-triyl)triacetic acid
