# Supplementary material for: Tetraspanin 8 (TSPAN 8) as a potential target for radio-immunotherapy of colorectal cancer
Source: Oncotarget. 2017 Feb 28;8(13):22034–47. doi: 10.18632/oncotarget.15787 (PMC5400644; doi:10.18632/oncotarget.15787)
Supplement: Supplementary file 1 [file oncotarget-08-22034-s001.pdf]

Tetraspanin 8 (TSPAN 8) as a potential target for radio-immunotherapy of colorectal cancer

SUPPLEMENTARY FIGURES AND TABLES

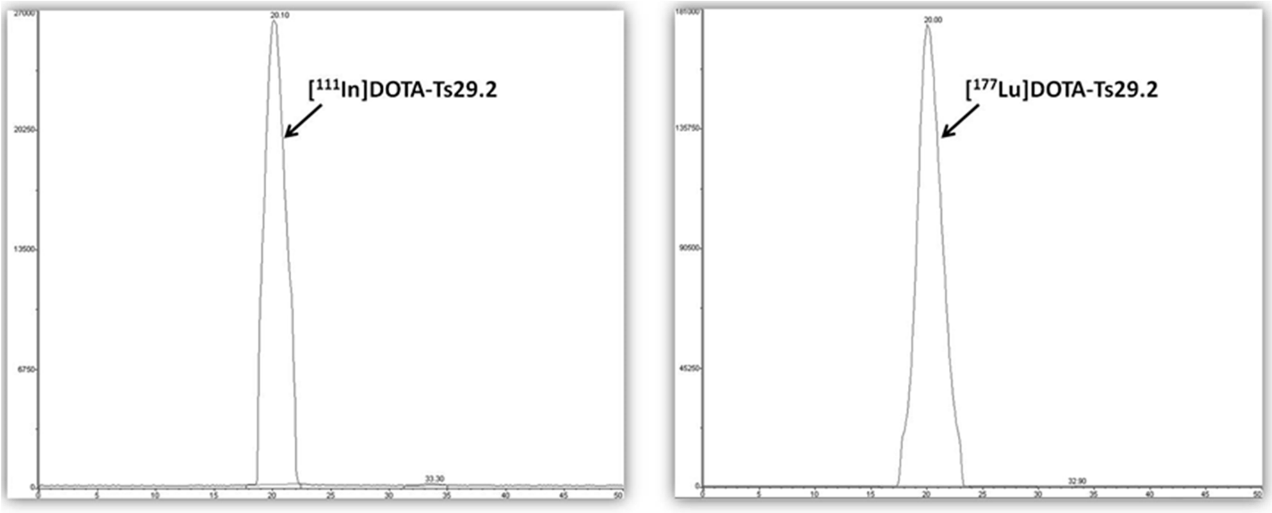

Analytic size exclusion HPLC analyses performed on a Superose 12 column, 10/300 GL, 11 µm (GE Healthcare), isocratic elution (20 mM 4-(2-hydroxyethyl)-1-piperazineethanesulfonic acid HEPES, 150 mM NaCl, pH = 7.3, flow rate: 0.5 mL.min^{-1})

Supplementary Figure 1: HPLC analyses of radiolabeled of [^{111}In]DOTA-Ts29.2 (left) and [^{177}Lu]DOTA-Ts29.2 (right).

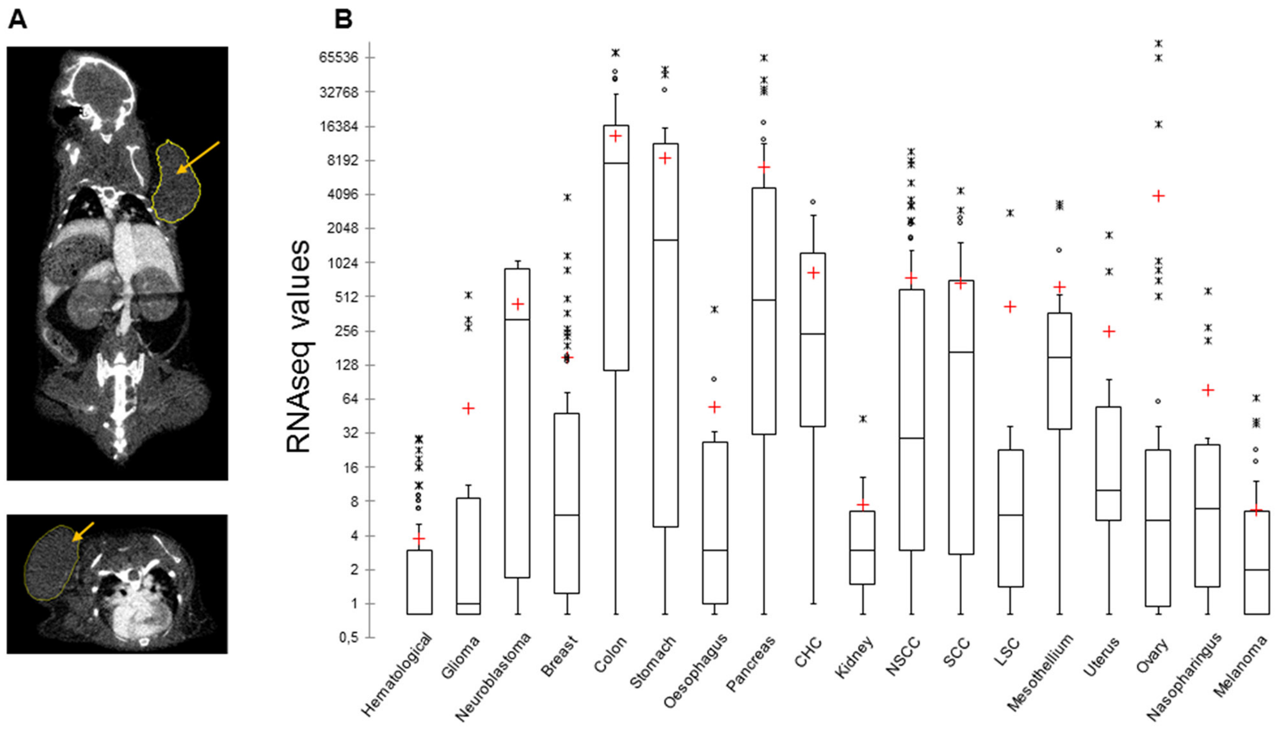

Supplementary Figure 2: A. CT images (left: sagittal, right: axial) of mice used for volume organs determination involved in S factors calculation. The arrows indicate the tumors. **B.** mRNA quantification of TSPAN8 in different cell lines according to the tissue type. These data were extracted from the study of Klijn C. *et al*. [35]. Red crosses represent the mean, box plots encompass the median value (central line), and crosses that do not enter in the boxes can be considered as extreme values.

Supplementary Tables 1 and 2: Dosimetry calculations performed by computing S factors with the GATE Monte Carlo platform and biodistribution data

^{177}Lu S-factors		targets							
Gy/Bq/s		rest of body	tumor	spleen	kidneys	liver	heart	lungs	brain
sources	*rest of body*	**6.06E-13**	3.13E-14	8.62E-14	3.85E-14	5.00E-14	4.91E-14	5.80E-14	3.82E-14
	tumor	3.65E-14	**3.21E-11**	1.54E-17	1.88E-17	6.06E-17	5.16E-17	2.02E-16	1.97E-17
	spleen	1.01E-13	1.52E-17	**1.62E-10**	6.18E-14	2.65E-15	2.67E-17	4.64E-17	5.21E-18
	kidneys	4.52E-14	1.80E-17	6.72E-14	**3.95E-11**	3.23E-14	1.89E-17	2.96E-17	3.45E-18
	liver	5.80E-14	5.94E-17	2.61E-15	3.41E-14	**1.47E-11**	5.18E-15	8.45E-14	7.17E-18
	heart	4.89E-14	5.95E-17	2.47E-17	1.70E-17	1.22E-14	**4.66E-11**	3.80E-12	1.79E-17
	lungs	6.74E-14	2.04E-16	4.64E-17	2.91E-17	8.44E-14	9.84E-13	**3.78E-11**	1.85E-17
	brain	4.43E-14	1.97E-17	5.09E-18	3.30E-18	7.89E-18	2.19E-17	1.89E-17	**5.21E-11**

^{90}Y S-factors		targets							
Gy/Bq/s		rest of body	tumor	spleen	kidneys	liver	heart	lungs	brain
sources	*rest of body*	**4.71E-12**	5.37E-13	2.91E-12	2.41E-12	2.18E-12	1.80E-12	2.40E-12	1.75E-12
	tumor	1.42E-12	**8.51E-11**	5.24E-16	1.61E-14	3.48E-12	2.04E-15	2.39E-12	2.81E-16
	spleen	3.35E-12	2.11E-16	**5.50E-11**	3.88E-11	3.00E-13	3.56E-16	5.17E-16	8.57E-17
	kidneys	3.85E-12	2.68E-16	7.92E-13	**7.52E-11**	1.14E-13	2.80E-16	3.86E-16	7.35E-17
	liver	3.01E-12	4.52E-15	1.91E-12	2.36E-11	**2.64E-11**	7.45E-14	2.07E-13	1.26E-16
	heart	1.41E-12	1.34E-15	9.11E-16	6.16E-16	3.51E-11	**1.15E-10**	1.63E-11	2.77E-16
	lungs	1.89E-12	2.69E-13	1.79E-12	1.08E-10	4.10E-11	1.22E-11	**4.17E-11**	2.54E-16
	brain	3.82E-12	1.80E-15	1.76E-16	1.32E-16	2.92E-16	1.29E-15	9.44E-16	**1.01E-10**

Dose values (Gy) were calculated for a 3.7 MBq injection of the radiolabeled antibody Ts29.2. Ratios of doses between tumor and other organs were evaluated
